# Estimating the axial strain of circular short columns confined with CFRP under centric compressive static load using ANN and GRA techniques

**DOI:** 10.1016/j.heliyon.2024.e34146

**Published:** 2024-07-04

**Authors:** Ammar T. Al-Sayegh, Nasim Shakouri Mahmoudabadi, Lamis J. Behbehani, Saba Saghir, Afaq Ahmad

**Affiliations:** a Department of Civil Engineering, College of Engineering & Petroleum, Kuwait University, Kuwait; b Department of Civil Engineering, The University of Memphis, TN, USA; c Department of Communication Design & Interiors, College of Architecture, Kuwait University, Kuwait

**Keywords:** Artificial neural networks, CFRP, Confined concrete, Regression analysis, Strain model

## Abstract

This investigation introduces advanced predictive models for estimating axial strains in Carbon Fiber-Reinforced Polymer (CFRP) confined concrete cylinders, addressing critical aspects of structural integrity in seismic environments. By synthesizing insights from a substantial dataset comprising 708 experimental observations, we harness the power of Artificial Neural Networks (ANNs) and General Regression Analysis (GRA) to refine predictive accuracy and reliability. The enhanced models developed through this research demonstrate superior performance, evidenced by an impressive R-squared value of 0.85 and a Root Mean Square Error (RMSE) of 1.42, and significantly advance our understanding of the behavior of CFRP-confined structures under load. Detailed comparisons with existing predictive models reveal our approaches' superior capacity to mimic and forecast axial strain behaviors accurately, offering essential benefits for designing and reinforcing concrete structures in earthquake-prone areas. This investigation sets a new benchmark in the field through meticulous analysis and innovative modeling, providing a robust framework for future engineering applications and research.

## 1 Introduction

Nowadays, concrete structures located near seismic areas are increasingly being strengthened and rehabilitated with fiber-reinforced polymers (FRPs). The main cause for preferring FRPs usage is enhancement in the axial and flexural strengths of concrete columns and structural elements, respectively [1]. One of the most popular uses of FRPs is the strengthening, repairing, and enhancement of ductility for reinforced concrete (RC) columns under corrosive and aggressive environments. The FRP composites are durable, lightweight, corrosion-resistant, electromagnetic-resistant, chemical-resistant, and have high tensile strengths [2,3]. The FRPs are the most suitable materials to be used in marine structures and coastal environments due to their high corrosion resistance property [4]. These composites are very efficient in providing the confinement mechanism. The confinement mechanism depends upon their thickness, elastic modulus, the number of wraps, strength of confined material, and the angle of orientation wrap to the structural element. FRPs have become the most efficient and popular way to reinforce structural concrete by lateral confinement [[5], [6], [7], [8]]. The FRPs are favored over steel jackets due to the advantages of reduced construction time, easy installation, easy handling, and slight disturbance of structural elements.

Concrete members' ductility and strength are mainly enhanced by the strengthening technique used for FRP-confined concrete structural elements [9,10]. In order to improve the strength and ductility of concrete structures affected by the earthquake, they will need to be rehabilitated and retrofitted [11]. FRP wraps improve the axial strength of bridge piers by reducing lateral movement. The FRP confinement prevents the lateral expansion of core material, which is the main reason for the strength and ductility enhancement of concrete elements. This lateral confinement mechanism is more efficient when a triaxial stress environment is provided to concrete core material. Compared with the confinement of steel ties or spirals, FRPs provide continuous confinement action for the whole cross-section of the members, along with ease of installation [12]. The confinement effect due to FRPs depends on the shape of the confined concrete structural member. This effect is more efficient for the structural members with a minimum radius of corners.

Recent studies have highlighted remarkable advancements in both theoretical and practical realms within the fields of structural engineering and materials science, signaling a transformative period for these disciplines [[13], [14], [15]]. These investigations collectively contribute to the development of infrastructures that are more resilient and capable of withstanding various environmental challenges [16,17]. Notably, the application of advanced computational techniques, such as Artificial Neural Networks (ANN), represents a significant leap toward sustainable construction practices [18]. This corpus of research exemplifies the interdisciplinary approach required to tackle modern engineering challenges and underscores a concerted global effort to foster smarter, safer, and more ecologically sustainable structural solutions [[19], [20], [21]]. Furthermore, the integration of artificial intelligence algorithms for predicting material strength is a testament to the innovative strategies employed to enhance building materials' reliability and efficiency [22].

In the study [[23], [24], [25], [26]], metaheuristics-based artificial neural networks were developed to predict the confinement effect of CFRP on concrete strength, while Shakouri Mahmoudabadi et al. [27] explored the effects of eccentric loading on concrete columns reinforced with GFRP bars, contributing to the broader understanding of concrete behavior under various loading conditions.

Even without knowing the type of interaction, ANN (artificial neural networks) tend to develop strange relationships between system variables, in which the assumptions underlying the mechanism are not taken into account [28]. Previously, ANNs were usually used to predict material response and member response [29]. In this study, ULR assessed the response of complex and simple reinforced concrete members using ANNs. For estimating the ULR of composite concrete members (CCM), artificial neural networks have received increased attention from researchers [30,31].

Recent advancements in predictive modeling for concrete's mechanical properties have significantly leveraged artificial neural networks (ANNs) and M5P-tree methodologies. Studies like those by Mohammed et al. [32] and Ahmed et al. [33] have successfully applied these techniques to predict compression strength in polymer-modified and geopolymer concretes. Similarly, Onyelowe et al. [34] and Reda et al. [35] have utilized AI to enhance strength predictions in CFRP-wrapped concrete structures. Kumar et al. [36,37] have further extended this approach to include FRP-concrete bond strength and carbonation depth estimations, illustrating the broad utility of machine learning in structural engineering applications, as seen in the work of Jusoh et al. [38] and Mohamad et al. [39] on precast tunnel linings and pile instrumentation.

In various structural engineering problems, the applications of Artificial Neural Networks (ANNs) are increasing [40,41]. Using ANNs, the complex interaction mechanisms between various variables and their behaviors can be determined without knowing the nature of the interactions. It is also possible to use ANNs to predict the compressive strength of confined plastic concrete. The axial compressive strength of FRP was predicted using the ANN technique by Reddy et al. [42] and Khademi et al. [43]. Carbon Fiber Reinforced Polymer (CFRP) wraps improved the compressive, tensile, and flexure strength of concrete members, and close agreement between experimental and theoretical results was observed [25,[44], [45], [46]]. Several works in the literature have explored the interaction and confinement mechanism of FRP wraps and proposed new mathematical models for axial strengths and strain prediction using regression analysis, ANNs, curve fittings, genetic programming [47], and soft computing [48,49]. These models were based on fewer experimental sample points, using simple spreadsheets and curve fittings techniques, and having limited variables and functions to cover all the interaction and confinement mechanisms. It is still necessary to develop a new model to predict strains in FRP-confined concrete elements based on large experimental data sets that consider the various parameters and interaction mechanisms of FRP-confinement.

In developing sophisticated models for predicting axial strain in CFRP-constrained cylinders, insights from diverse fields are invaluable. Banaeipour et al. [50,51] explored the effects of fiber orientation deviations on compressive characteristics, while Ghasemi and Naser [52] investigated 3D-printed concrete tailoring using AI. Khoei et al. [53] modeled density-driven flow in reservoirs with fractures, and Manavi et al. [54,55] applied genetic algorithms and neural networks for cloud resource allocation, showcasing diverse methodologies applicable to CFRP modeling.

This study enhances the predictive modeling of axial strains in CFRP-confined concrete cylinders, which is crucial for designing and rehabilitating infrastructure in seismic areas [56]. Utilizing a comprehensive database of 708 experimental sample points, along with artificial neural networks (ANNs) and regression analysis, our research advances more accurate and reliable models for predicting the behavior of confined concrete [29]. The newly developed models, which demonstrate improved accuracy with superior RMSE and R^2^ values over existing models, accurately capture the axial compressive strains, offering more effective engineering solutions. Practical applicability of these models allows engineers and researchers to predict axial strains in real-world scenarios where CFRP confinement is used to enhance the ductility and strength of concrete structures. By inputting material properties, geometric parameters, and confinement characteristics into the models, practitioners can forecast the behavior of confined concrete under various load conditions, facilitating optimized design and safety assessments of structural elements. This application not only saves design time and reduces costs but also ensures the resilience of structures in earthquake-prone regions. The integration of this research into practical applications provides engineers with robust tools for designing safer, more durable structures and contributes to a broader understanding of composite materials in construction. This study addresses a significant gap in the literature and lays the groundwork for future research into the complex interactions of materials under confinement, potentially transforming approaches to global civil engineering challenges.

## 2 FRP confinement mechanics

**Fig. 1:**
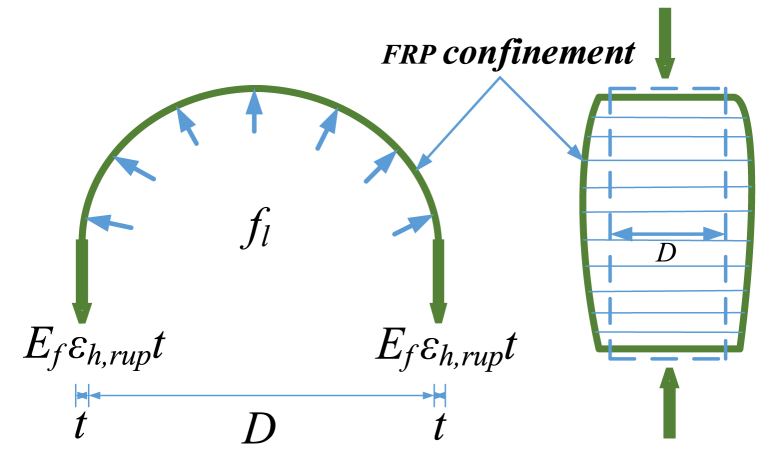
Confinement due to FRP wraps.

A low axial compressive load on the concrete compression members negates the lateral confinement effect. Nevertheless, when the load reaches the maximum strength of the members, the lateral confinement effect is activated to develop a lateral pressure that prevents the core material from dilating. The lateral confinement effect develops stress known as confinement stress, as shown in Fig. 1. Equation (1) can be used for the calculation of lateral confinement stress ($f_{l}$).

(1) $$f_{l}=\rho_{\varepsilon }\rho_{k}f_{\text{co}}^{\prime }$$

Where $\rho_{\varepsilon }$ and $\rho_{k}$ are strain ratio and stiffness ratio of confinement, respectively, and $f_{\text{co}}^{\prime }$ represents axial strength for unconfined concrete [57].

Equation (2) calculates the strain ratio ($\rho_{\varepsilon }$), which is a measure of how the strain capacity of the confined concrete compares to that of unconfined concrete. It is expressed as:

(2) $$\rho_{\varepsilon }=\frac{\varepsilon_{h,\text{rup}}}{\varepsilon_{\text{co}}}$$

Equation (3) defines the stiffness ratio ($\rho_{k}$), which evaluates the increase in stiffness due to confinement relative to the unconfined concrete's axial stiffness. This equation is given by:

(3) $$\rho_{k}=\frac{2E_{f}t}{\left(\frac{f_{\text{co}}^{\prime }}{\varepsilon_{\text{co}}}\right)D}$$

In these equations, ‘t’ represents the total thickness of FRs, $\varepsilon_{\text{co}}$ represents compressive unconfined concrete strain, $\varepsilon_{h,\text{rup}}$ is FRPs rupture strain in the lateral direction, and $E_{f}$ is FRP's Young's modulus. A relationship for this parameter was suggested in the previous work [58].

(4) $$\varepsilon_{h,\text{rup}}=\frac{\varepsilon_{f}}{f_{\text{co}}^{\prime 0.125}}$$

where, $\varepsilon_{f}$ represents the ultimate tensile strain for FRPs. By putting values for $\rho_{k}$ and $\rho_{\varepsilon }$ in Eq. (1), the relationship for $f_{l}$ take the form as given by the following relationship.

(5) $$f_{l}=\frac{2E_{f}\varepsilon_{h,\text{rup}}t}{D}$$

In Table 1, based on the developed database, all the famous strain models that were assessed, selecting the new model in a more general form, are listed.

**Table 1:** Previously proposed strain models.

Strain models	Expression
**Fardis and Khalili** [12]	$\varepsilon_{cc}=0.002+0.001\frac{E_{f}t}{Df_{co}^{\prime }}$
**Samaan et al.** [44]	$\varepsilon_{cc}=\frac{f_{cc}^{\prime }-f_{o}}{245.61{f_{co}^{\prime }}^{0.2}+1.3456\frac{E_{f}t}{D}}$${where\;f}_{o}=0.872f_{co}^{\prime }+0.371f_{l}+6.258$
**Karbhari and Gao** [59]	$\varepsilon_{cc}=\varepsilon_{co}+0.01\frac{f_{l}}{f_{co}^{\prime }}$
**Mander et al.** [60]	$\frac{\varepsilon_{cc}}{\varepsilon_{co}}=1+5\left(\frac{f_{cc}^{\prime }}{f_{co}^{\prime }}-1\right)$
**Toutanji** [61]	$\frac{\varepsilon_{cc}}{\varepsilon_{co}}=1+\left(310.57\varepsilon_{h,rup}+1.90\right)\left(\frac{f_{cc}^{\prime }}{f_{co}^{\prime }}-1\right)$
**Lam and Teng** [62]	$\frac{\varepsilon_{cc}}{\varepsilon_{co}}=1.75+12\rho_{k}\rho_{\varepsilon }^{1.45}$
**Teng et al.** [63]	$\frac{\varepsilon_{cc}}{\varepsilon_{co}}=1.75+6.5{\varepsilon_{k}^{0.8}\rho }_{\varepsilon }^{1.45}$

## 3 Artificial neural networks (ANNs)

**Fig. 2:**
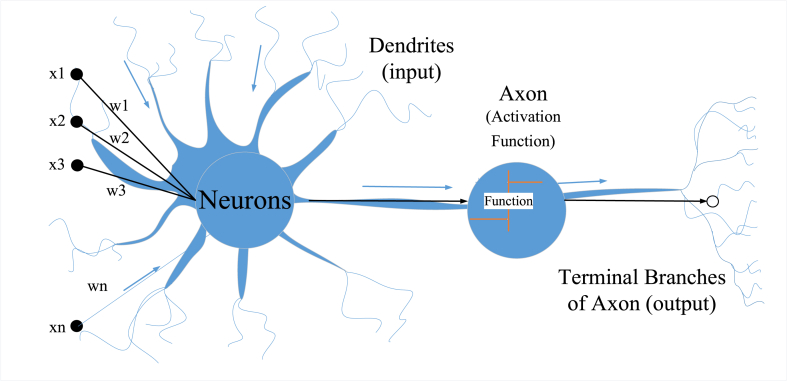
Structure of ANNs.

A neural network is a model of the human and animal nervous systems that function as the brain's critical information [64,65]. In this case, the networks are designed to evaluate purposes depending on a wide range of information provided. In addition to collecting and categorizing, ANNs can summarize and predict the given task. This is because the data added to their memory during the training phase could be saved, as well as their capacity to adapt. They consist of numerous connected layers, each composed of an interconnected neuron system. As shown in Fig. 5, in continuous layers, every two neurons have a tie (with a numerical value). These weights are then multiplied by the neuron's prediction. In this subsequent step, the neuron's prediction is passed across the link, adding to the bias, as illustrated in Fig. 5. Due to its roots in Multilayer Feedforward Networks (MLFNN), ANN (MLFNN) is regarded as the most appropriate for handling these types of issues. MLFNN also contains one or more hidden layers along with input (information) and output (target). As is interesting, these neurons are not tied to neutrons within the same layer but to neutrons in other layers. A variety of methods have been employed to minimize error values through several training cycles [[9], [10], [11]]. According to the author's previous work, input data is broken down into three segments, and the cross-validation method is applied in order to decrease error [[9], [10], [11]]. If this is the case, we can say that the network has become familiar with how a specific purpose works. The algorithm propagates errors from the output node back to the input node, as the name suggests. ANN's proposed architecture specifies the number of neurons in each layer(s) and also the quantity of hidden layers, as displayed in Fig. 2. In Eq (6), the artificial neuron is explained mathematically (see Fig. 6).

(6) $$O=f\left(\sum x_{z\;}w_{z}+b\right)$$

Where:

w_z_ are link values,

O gives the value predicted from ANN.

x_z_ are assigned inputs,

b are bias values (values of extra parameters).

The activation results are then transmitted to the following layer. The ultimate weights are computed based on the provided data. Researchers have used ANNs in past and present investigations to estimate load-carrying capacity for materials and individual RC members [66,67]. In this study, hyperbolic and tanh activation functions have been located between the input (information) and intermediary (processing) layers, whereas Intermediary (processing) and output (target) layers are only connected using hyperbolic functions. Eq (7) [68], can count the inaccuracy that occurred during the process. Eq (7) is employed for the comparison of outputs after ANN has been conditioned.

(7) $$E\left(w\right)=\frac{1}{2}\sum_{i\;}{\left[T-O\right]}^{2}$$

Where: O is predicted, and T is the target value.

**Fig. 3:**
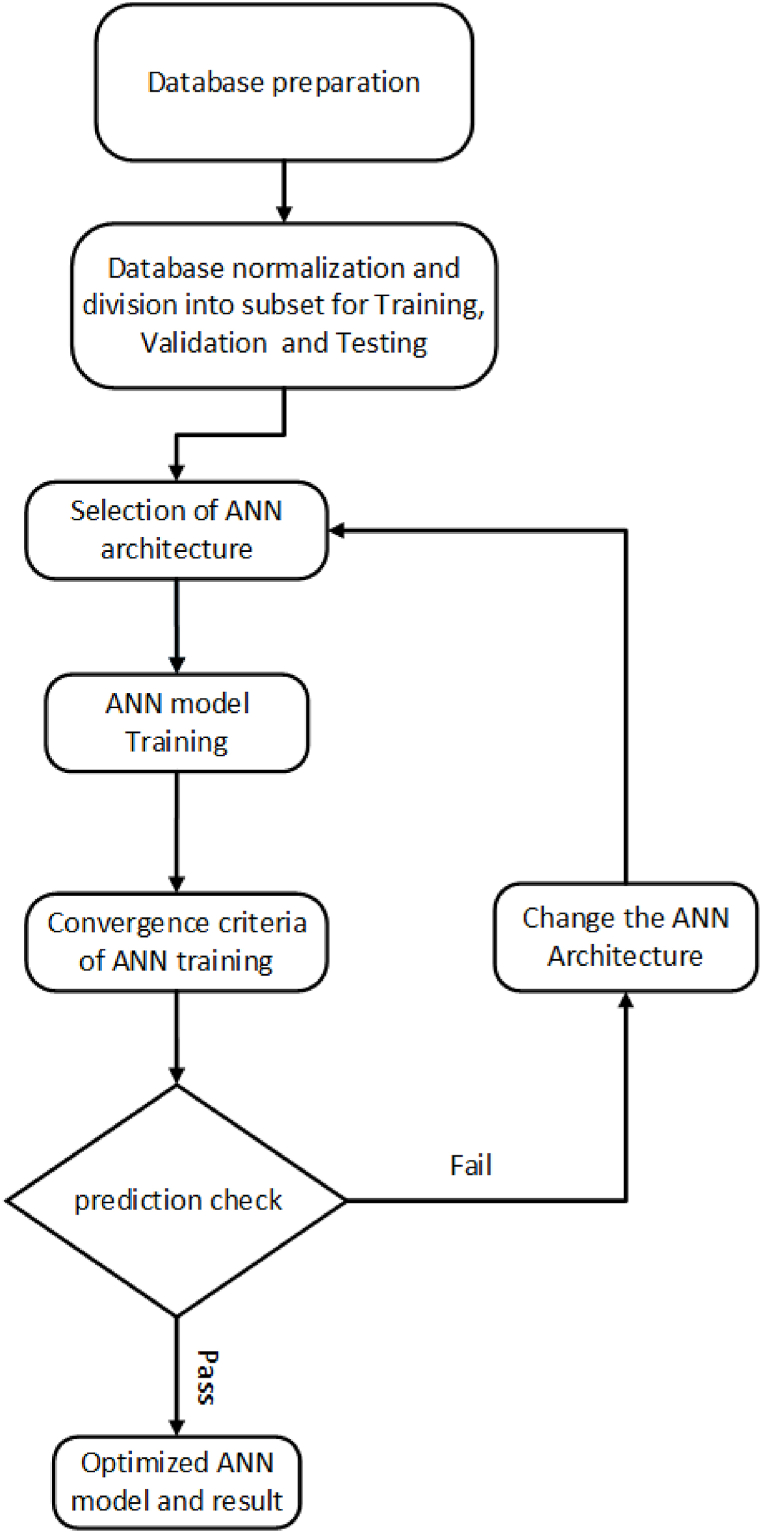
Developing process for ANNs.

Fig. 3 illustrates the development process of the ANN model.

**Fig. 4:**
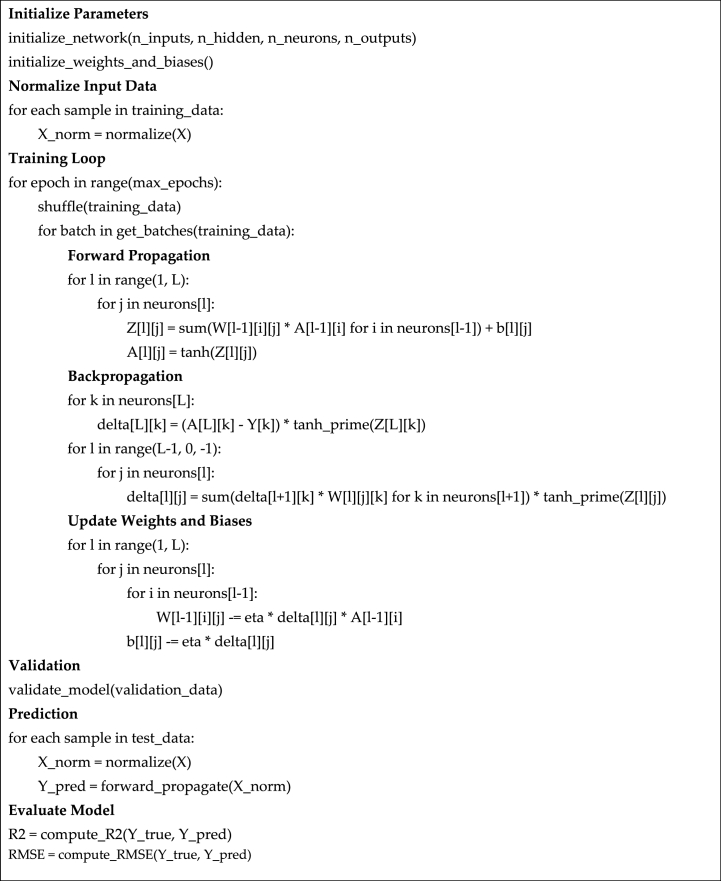
Developing process for ANNs.

The pseudo-code used to develop the ANN model is shown in Fig. 4.

**Fig. 5:**
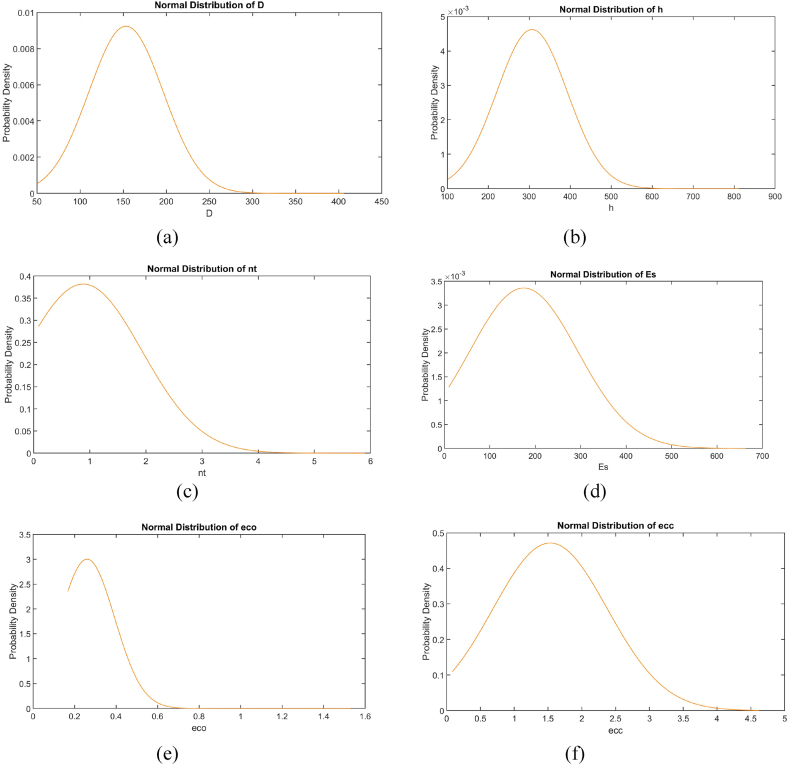
Normal distribution of (a) d (b) h (c) nt (d) $E_{f}$ (e) $\varepsilon_{co}$ (f) $\varepsilon_{cc}$ for FRP-confined specimens.

**Fig. 6:**
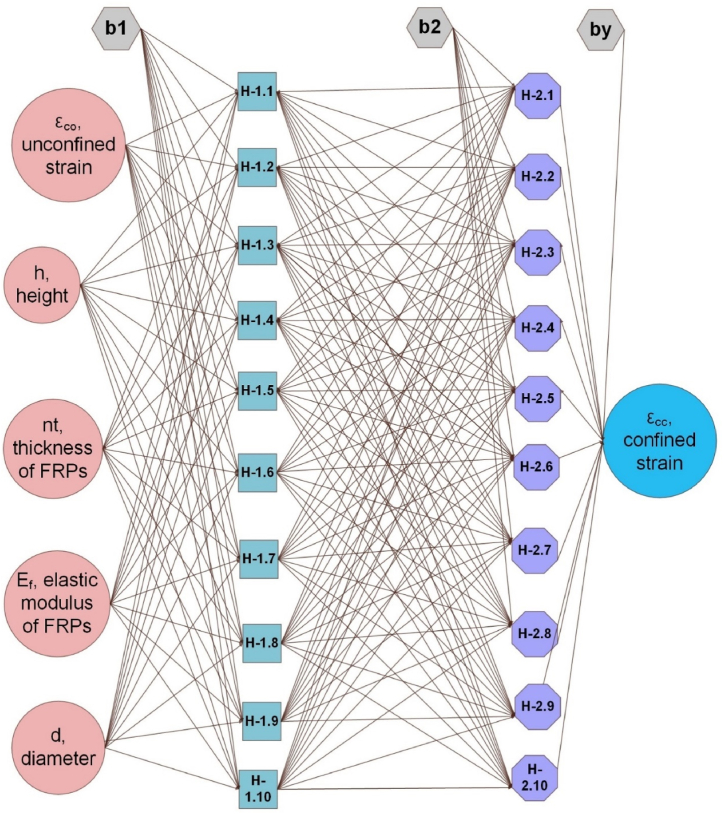
ANN model used in the present work.

### 3.1 Development of database

The database utilized in this study is a comprehensive compilation of 708 records. These records have been partitioned with 60 % used for training, 20 % for validation, and 20 % for testing [69]. This partitioning ensures a balanced approach to model training, validation, and testing, enhancing the robustness of our findings, each record consisting of multiple parameters, including diameter, height, and material properties relevant to the confinement of concrete cylinders with CFRP. The primary outputs from this dataset are the measured axial strain and compressive strength of these specimens. The data were sourced from an extensive review of the literature, selecting studies that provided detailed experimental results on CFRP-confined concrete cylinders. These studies are specifically referenced throughout our manuscript, linking their findings directly to our analytical models. In developing this robust database, we included all factors that can potentially affect the axial compressive strain in concrete members confined with FRP, enhancing the database's breadth and applicability. It is important to note that some sample points initially included in the database were later removed due to their poor predictions and potential to skew the root mean square error (RMSE). This decision was made to ensure the accuracy and reliability of our analysis, avoiding the saturation of RMSE values and maintaining the integrity of the data. Detailed statistical information about each parameter, including height and diameter, has been meticulously recorded and is presented in Table 2, Table 3, Table 4, Table 5.

**Table 2:** The statistical analysis of the whole database.

Parameter	Unit	Min	Max	Diff	Avg	St. Dev	COV
***D***	(mm)	51	406	355	153.33	43.15	0.28
***H***	(mm)	102	812	710	306.86	86.32	0.28
nt	(mm)	0.09	5.9	5.81	0.88	1.04	1.18
$E_{f}$	MPa	10	663	653	174.68	118.8	0.68
$\varepsilon_{co}$	(%)	0.1676	1.53	1.3624	0.26	0.13	0.5
$\varepsilon_{cc}$	(%)	0.083	4.62	4.537	1.53	0.85	0.56

**Table 3:** The statistical analysis of the training database.

Parameter	Unit	Min	Max	Diff	Avg	St. Dev	COV
*D*	(mm)	51	406	355	151.71	42.3	0.28
*H*	(mm)	102	812	710	303.57	84.56	0.28
nt	(mm)	0.09	5.9	5.81	0.87	1.04	1.2
$E_{f}$	MPa	10	663	653	180.8	119.99	0.66
$\varepsilon_{co}$	(%)	0.1676	1.53	1.3624	0.26	0.14	0.54
$\varepsilon_{cc}$	(%)	0.083	4.62	4.537	1.54	0.85	0.55

**Table 4:** The statistical analysis of the validating database.

Parameter	Unit	Min	Max	Diff	Avg	St. Dev	COV
*D*	(mm)	51	406	355	155.01	45.93	0.3
*H*	(mm)	102	812	710	311.25	91.54	0.29
nt	(mm)	0.11	4.28	4.17	0.87	0.95	1.09
$E_{f}$	MPa	19	612	593	174.05	115.78	0.67
$\varepsilon_{co}$	(%)	0.1676	1.33	1.1624	0.26	0.12	0.46
$\varepsilon_{cc}$	(%)	0.126	3.66	3.534	1.6	0.87	0.54

**Table 5:** The statistical analysis of the testing database.

Parameter	Unit	Min	Max	Diff	Avg	St. Dev	COV
*D*	(mm)	70	406	336	156.52	42.88	0.27
*H*	(mm)	140	812	672	312.36	86.29	0.28
nt	(mm)	0.11	5	4.89	0.96	1.14	1.19
$E_{f}$	MPa	10	438	428	156.87	117.18	0.75
$\varepsilon_{co}$	(%)	0.1676	1.28	1.1124	0.26	0.13	0.5
$\varepsilon_{cc}$	(%)	0.103	4.36	4.257	1.44	0.8	0.56

Where.

**Table:** 

*D*:	Diameter
*H*:	Height
nt:	Thickness of FRP Wrap
$E_{f}$:	Elastic Modulus of FRP
$\varepsilon_{co}\text{:}$	Strain of Unconfined Concrete
$\varepsilon_{cc}$:	Strain of CFRP-Confined Concrete

The normal distribution curves for the input and output parameters, displayed in Fig. 5, provide a visual representation of the probability density functions, ensuring that the data covers all the ranges considered.

All the architectures of the ANNs being applied in the current study are described in Table 6. Six different ANN models were created in the current work, varying in activation functions, neurons, and hidden layers.

**Table 6:** The architecture of various ANN models in the present study.

Models	Inputs	AF between IL and HL	Number of neurons in HL	AF between HL and OL	Output
**ANN**_**1**_	d, h, t, $E_{f}$, $\varepsilon_{\text{co}}$	Sigmoid	5	Tanh	$\varepsilon_{\text{cc}}$
**ANN**_**2**_	d, h, t, $E_{f}$, $\varepsilon_{\text{co}}$	Sigmoid	10	Tanh	$\varepsilon_{\text{cc}}$
**ANN**_**3**_	d, h, t, $E_{f}$, $\varepsilon_{\text{co}}$	Sigmoid	5–5	Tanh	$\varepsilon_{\text{cc}}$
**ANN**_**4**_	d, h, t, $E_{f}$, $\varepsilon_{\text{co}}$	Sigmoid	10–10	Tanh	$\varepsilon_{\text{cc}}$
**ANN**_**5**_	d, h, t, $E_{f}$, $\varepsilon_{\text{co}}$	Sigmoid	5-5-5	Tanh	$\varepsilon_{\text{cc}}$
**ANN**_**6**_	d, h, t, $E_{f}$, $\varepsilon_{\text{co}}$	Sigmoid	10-10-10	Tanh	$\varepsilon_{\text{cc}}$

### 3.2 Normalization of constructed database

Due to the unitless quantities of the inputs so far, the standard data points procedure will have a major influence on ANN learning [42]. The inputs were transformed, stretching between 0.1 and 0.9.; 0.1 being lower and 0.9 upper limits, respectively, by using Eq. (8).

(8) $$X=\frac{\Delta X}{\Delta x}x+\left(X_{\max}-\frac{\Delta X}{\Delta x}x_{\max}\right)$$

Where:

X represents the normalized/uniform value, x indicates the current value; and Δ denotes the ratio of limits. Specifically, the value of the X_max_ = 0.9 and ΔX = 0.8 are compared with the new ratio of 0.1–0.9.

Pearson's correlation coefficient (R) values were evaluated using Eq (9) [30,31,40,42]. This coefficient measures the linear correlation between two variables, target, and output, providing insight into the strength and direction of their linear relationship. A higher absolute value of ∣R∣ indicates a stronger linear relationship between the input and output parameters, establishing the relationship's significance. If the calculated ∣R∣ for a particular input-output pair is relatively high, it underscores the influential role of that input parameter on the output, potentially guiding further analysis and model refinement.

(9) $$R=\frac{\sum_{i=1}^{n}\left[\left(T_{i}-\bar{T}\right)\left(O_{i}-\bar{O}\right)\right]}{\sqrt{\sum_{i=1}^{n}{\left(T_{i}-\bar{T}\right)}^{2}\cdot \sum_{i=1}^{n}{\left(O_{i}-\bar{O}\right)}^{2}}}$$

ANN models were modeled and trained according to the author's own procedures and guidelines [30,31,40,42].

In MATLAB, a multilayer ANN model was employed for the training process of the ANN, using Levenberg-Marquardt with MLFNN method [43]. The architect of the ANN was adopted from Pham and Ashrafi's work [31,40]. The selection of the optimal ANN model was performed on (i) The error calculated by Pearson's correlation coefficient (R), (ii) Mean Squared Error (MSE), and (iii) Mean Absolute Error (MAE) [44] Eqs. (9), (10), (11)) shows the analytical expression the mentioned parameters.

(10) $$MSE=\frac{\sum_{i=1}^{n}{\left(T_{i}-O_{i}\right)}^{2}}{n}$$

(11) $$MAE=\frac{\sum_{i=1}^{n}\left|T_{i}-O_{i}\right|}{n}$$

**Fig. 7:**
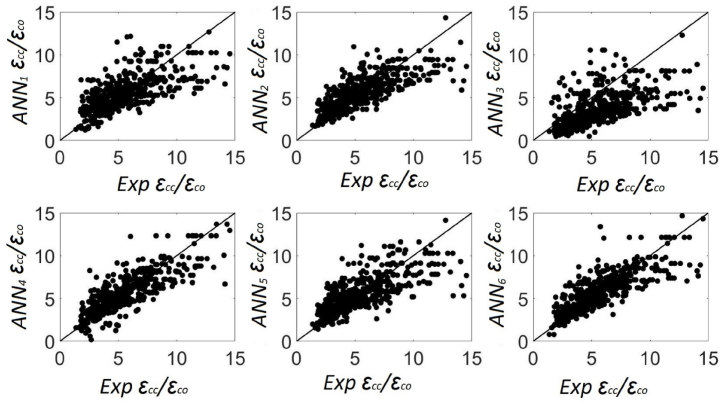
Predictions of proposed six ANN models.

**Fig. 8:**
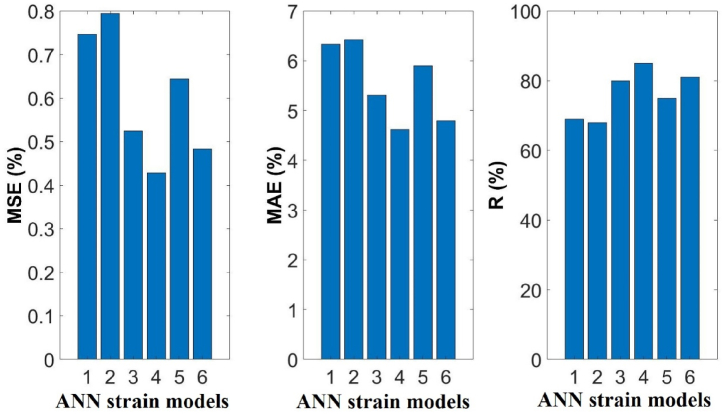
Statistical error variables presented by proposed ANN models.

Where: Oi are the specified target results, and Ti are anticipated results from ANN; the ratio of test values (Ti) and anticipated values (Oi), and n is the total quantity of samples in the databank. The optimized ANN model yielded the highest R values with the corresponding lowest MAE and MSE [[9], [10], [11]].

n represents the number of points in the sample for these equations, $T_{i}$ refers to the testing value obtained from experiments, $O_{i}$ is the value predicted by the ANN models, $\bar{T}=\sum_{1}^{n}T_{i}/n$ is the average value of $T_{i}$, and $\bar{O}=\sum_{1}^{n}O_{i}/n$ is the average value of $O_{i}$. The statistical details of the estimations made by six ANN models and the experimental measurements are presented in Table 7. Moreover, the predictions and performances of ANN models for $\varepsilon_{\text{cc}}/\varepsilon_{\text{co}}$ were presented in Fig. 7, and the statistical error functions (MSE, MAE, and R) of the predictions of ANN models were presented in Fig. 8. The ANN model was selected as the most optimal model, and it presented the minimum values of R, MSE, and MAE [29]. The ANN_4_ portrayed a value of 85 % for R, 4.62 % for MAE, and 0.42 % for MSE, which were the best predictions. Therefore, ANN_4_ was selected as the most reliable ANN model for predicting the axial compressive strain of FRP-confined concrete members.

**Table 7:** The values of $\varepsilon_{\text{cc}}/\varepsilon_{\text{co}}$ for various ANN models.

Model	Min	Max	Diff	Avg	St. Dev	COV
***Exp***$\varepsilon_{\text{cc}}/\varepsilon_{\text{co}}$	1.375	17.2727	15.8977	5.56	2.73	0.49
**ANN**_**1**_	1.223	13.5319	12.3087	5.48	2.08	0.38
**ANN**_**2**_	1.650	14.3371	12.6871	5.58	2.07	0.37
**ANN**_**3**_	0.469	12.2890	11.8197	3.70	2.08	0.56
**ANN**_**4**_	0.171	16.0476	15.8762	5.58	2.50	0.45
**ANN**_**5**_	1.411	14.1429	12.7323	5.58	2.13	0.38
**ANN**_**6**_	−0.818	14.6667	15.4848	5.59	2.38	0.43

## 4 Evaluation of various strain models

Using the developed database, the design-oriented strain model was suggested in this work. The statistical functions (sum of square errors (SSE), R^2,^ and RMSE) were used to assess previous strain models in the database and select a new model with the best structure. Assessment of the models suggested by Karbhari and Gao [59], Fardis and Khalili [12], Lam and Teng [62], Mander et al. [60], Toutanji [61], Samaan et al. [44], and Teng et al. [63] were made. The selection of these equations was done because of their wide applications in the scientific community. The parameters R^2^ and RMSE can be obtained from Eq. (12) and Eq. (13) correspondingly.

(12) $$R^{2}={\left(\frac{n\left(\sum xy\right)-\left(\sum x\right)\left(\sum y\right)}{\sqrt{\left[n\sum x^{2}-{\left(\sum x\right)}^{2}\right][n\sum y^{2}-{\left(\sum y\right)}^{2}}}\right)}^{2}$$

(13) $$RMSE=\sqrt{\frac{\sum {\left(x-y\right)}^{2}}{n}}$$

**Fig. 9:**
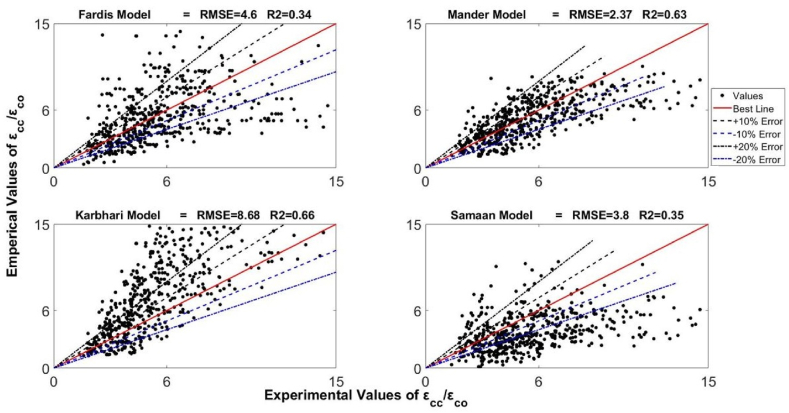

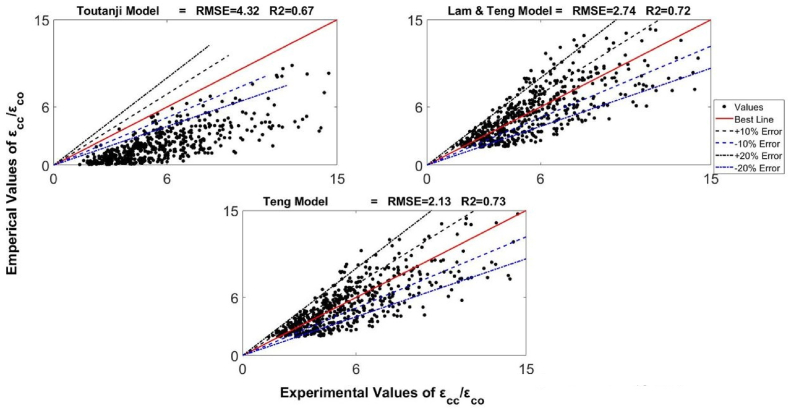
Performance of various strain models over-developed database

In these equations, x is the experimental measurements, the total number of tests equals n in the database, and y are the theoretical predictions of the axial compressive strains of FRP. As the value of RMSE moves towards zero, the accuracy increases. Conversely, When R^2^ moves towards one, prediction reliability rises. The best was secured by minimizing the error between experimental and theoretical results using SSE. As the value of SSE moves towards zero, the accuracy of predictions increases, showing the best fit at zero and no relationship at one. Based on R^2^ and RMSE, the performances of various strain models are shown in Fig. 9.

As exhibited in Fig. 9, the best performance among all of the studied models was presented by the equation proposed by Teng et al. [63]. This equation gave the values of 0.73 and 2.13 for R^2^ and RMSE, respectively. Accordingly, the form of the newly developed equation has been kept fairly close to the form which corresponded to the model proposed by Teng et al. [63] as represented by Eq. (14).

(14) $$\varepsilon_{cc}/\varepsilon_{co}=\alpha +\beta \rho_{k}^{\lambda_{1}}\rho_{\varepsilon }^{\lambda_{2}}$$

where α, β, $\lambda_{1}$ and $\lambda_{2}$ are the coefficients. After incorporating all the constant values that have been obtained from fitting the curve using the MATLAB program, Eq. (15), which is the newly proposed nonlinear model in this study, is obtained.

(15) $$\varepsilon_{cc}/\varepsilon_{co}=1.85+7.46\rho_{k}^{0.71}\rho_{\varepsilon }^{1.171}$$

**Fig. 10:**
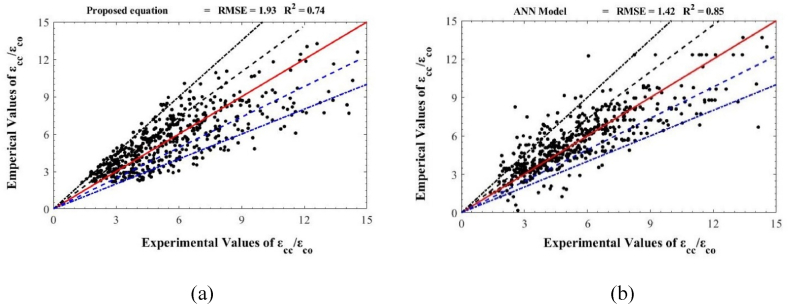
Performance of (a) proposed empirical strain model (b) proposed ANN strain models.

Fig. 10 illustrates the comparative performances of our newly developed empirical and ANN models in predicting the axial strains of CFRP-confined concrete. The empirical model (Fig. 10a) achieves an R-squared value of 0.74 and an RMSE of 1.93, while the ANN model (Fig. 10b) outperforms with an R-squared of 0.85 and an RMSE of 1.42. These statistics demonstrate the ANN model's superior accuracy and tighter data clustering around the predictive line, confirming its enhanced predictive capability over both the empirical model and previous approaches.

**Fig. 11:**
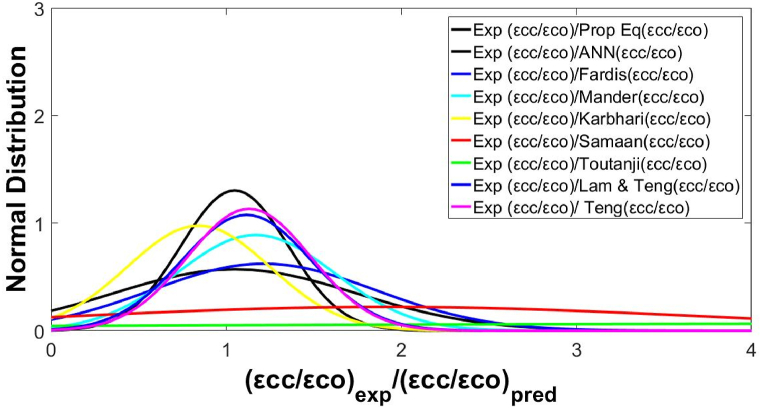
Normal distribution of $\left({\varepsilon_{cc}/\varepsilon_{co}}_{\exp}\right)/\;\left({\varepsilon_{cc}/\varepsilon_{co}}_{pred}\right)$ for FRP-confined specimens.

**Fig. 12:**
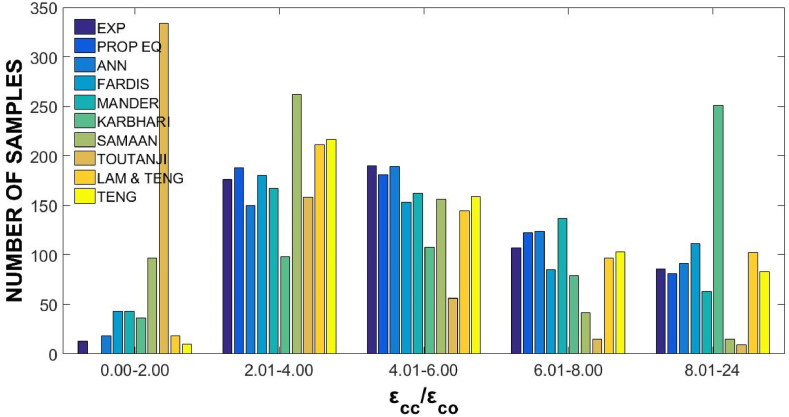
Distribution of $\varepsilon_{cc}/\varepsilon_{co}$ for FRP-confined specimens.

To further assess the reliability of the proposed models, the experimental predicted values and the normal distribution of FRP axial strains were displayed. These values comparisons were done with those taken from other models. Fig. 11 shows normal distribution curves of experimental to predicted axial strain ratios, and Fig. 12 shows the distribution of the experimental and predicted FRP ratios from confined to unconfined concrete strains. The Lam and Teng [62] model portrayed a better normalization process performance than the previous models.

Statistical analyses were rigorously conducted to assess the consistency of the data. For each dataset, the coefficient of variation and standard deviation were computed, substantiating the low variability and enhancing the credibility of the results across multiple tests. The experimental setup was meticulously standardized, including uniform use of materials and methodologies, to ensure consistent testing conditions across all experiments. This methodological consistency is pivotal for the reproducibility of our findings. Moreover, our results were systematically compared with extant data from analogous studies, demonstrating significant congruence and thereby reinforcing the validity of our models within the scientific community at large. We also explored the ramifications of any deviations observed, providing an exhaustive analysis of our models' performance under varied conditions. A critical evaluation of potential sources of error was performed, with a particular focus on their influence on our results. Future studies will implement strategies to mitigate these errors, thereby augmenting the accuracy and reliability of the experimental outcomes.

## 5 Conclusions

The current work performs a reliability analysis of the strain models proposed using artificial neural networks, finite element analysis, and general regression analysis on the basis of a large database of 708 testing results. This study could draw the following key points.

• The evaluation of currently proposed empirical strain model, supported by an extensive dataset of 708 tests, demonstrates a significant improvement in accuracy compared to earlier FRP-confined concrete strain models. This model achieved a root mean square error (RMSE) of 1.93 and an R-squared value (R^2^) of 0.74. In parallel, our advanced artificial neural network (ANN) model outperformed previously suggested models, recording an RMSE of 1.42 and an R^2^ of 0.85. These statistical metrics affirm the enhanced predictive capabilities of our current models over their predecessors.
• The testing results depicted that the axial compressive strength and strains of CFRP-confined concrete cylinders increased dramatically as a result of the confinement effect. The average enhancements of 368.75 % and 437.03 % occurred for the axial strains of the cylinder specimens. As regards the axial strength, the average improvements of 95.20 % and 65.12 % occurred within the axial compressive strengths of CFRP-confined concrete members.
• A critical assessment of the discrepancies between the empirical test results and model predictions reveals that the error percentages for the empirical and ANN models stand at 13.51 % and 10.68 %, respectively. Despite these discrepancies, the error margins are considered acceptable within the context of complex predictive modeling and support the proposed models' reliability. These findings not only validate the models' accuracy but also enhance our understanding of CFRP confinement dynamics in concrete cylinders.
• In predictive modeling, understanding the impact of various input parameters on the predicted outcome is crucial [70]. This sensitivity analysis aims to determine the influence of each input parameter on the axial strain of CFRP-confined concrete columns, considering parameters such as the diameter (D), height (H), thickness of the CFRP wrap (nt), elastic modulus of the CFRP (Ef), and strain of unconfined concrete (εco). The analysis was planned to be conducted using a trained Artificial Neural Network (ANN) model, involving parameter perturbation to isolate individual effects and measure the change in predicted axial strain (εcc) for each parameter variation. The expected results would show how increasing the diameter generally decreases axial strain, while height variations might have a moderate impact. It is anticipated that thicker CFRP wraps would significantly reduce axial strain, and higher elastic modulus values would result in lower axial strains, indicating stiffer materials are more effective in confinement. The initial strain of unconfined concrete is also expected to substantially affect the confined strain. The analysis aims to highlight that the thickness of the CFRP wrap and the elastic modulus of the CFRP are likely the most influential parameters, underscoring their importance in the design and retrofitting of concrete structures. Conducting this sensitivity analysis will enhance the robustness of predictive models and provide practical insights for optimizing the design of CFRP-confined concrete structures.
• Despite the advancements presented in this study, it is important to acknowledge several limitations. The empirical and ANN models developed are primarily based on a dataset derived from controlled laboratory conditions, which may not capture the complex variabilities encountered in real-world applications. The models also assume material homogeneity and ideal bonding conditions between the concrete and CFRP, assumptions that might not hold in practical scenarios. Furthermore, the reliance on controlled experimental data limits the generalizability of the findings. Future work should aim to validate these models under a broader range of environmental conditions and with varied concrete compositions. Additionally, investigating the impact of non-ideal bonding conditions will be crucial. Addressing these limitations will refine the models to better suit practical applications, enhancing their robustness and applicability across different operational environments.

## Funding

This research received no external funding.

## CRediT authorship contribution statement

**Ammar T. Al-Sayegh:** Writing – original draft, Methodology, Formal analysis. **Nasim Shakouri Mahmoudabadi:** Resources, Formal analysis, Data curation. **Lamis J. Behbehani:** Writing – original draft, Software, Resources. **Saba Saghir:** Visualization, Validation, Supervision, Resources. **Afaq Ahmad:** Writing – original draft, Formal analysis, Data curation.

## Declaration of competing interest

The authors whose names are listed in this paper certify that they have NO affiliations with or involvement in any organization or entity with any financial interest (such as honoraria; educational grants; participation in speakers’ bureaus; membership, employment, consultancies, stock ownership, or other equity interest; and expert testimony or patent-licensing arrangements), or non-financial interest (such as personal or professional relationships, affiliations, knowledge or beliefs) in the subject matter or materials discussed in this manuscript.
